# How gender norms and interpersonal communication are associated with gender-based violence attitudes and intentions to intervene: Secondary outcome findings from Odisha, India

**DOI:** 10.1177/22799036251395256

**Published:** 2025-11-20

**Authors:** Rajiv N. Rimal, Rohini Ganjoo, Daryl Stephens, Erica Sedlander

**Affiliations:** 1 Johns Hopkins University, Baltimore, MD, USA; 2 George Washington University, Washington, DC, USA; 3 University of California, San Francisco, CA, USA

**Keywords:** gender norms, interpersonal communication, gender-based violence, social norms

## Abstract

**Objectives:**

Despite high rates of gender-based violence in India, there are few theory-based interventions designed to reduce their incidence. In a secondary analysis of the data, we raised research questions pertaining to how harmful gender norms are perpetuated through interpersonal communication and their combined effects on attitudes about gender-based violence and intentions to intervene.

**Design and methods:**

In a cluster randomized controlled trial, longitudinal data were collected at baseline (*N* = 2048) and end-line (*N* = 3797) from women of reproductive age (15–49 years old) through one-on-one interviews in the eastern state of Odisha in India. The usual-care control group was only monitored, without the intervention, whereas the treatment group received a 3-year intervention as part of the Reduction in Anemia through Normative Innovations (RANI) Project. This paper uses only the end-line data.

**Results:**

Overall effect sizes were small, but significant interactions emerged in the relationships between norms and attitudes and between norms and intentions, with interpersonal communication and intervention effects as moderators. Strongest intentions were found in treatment communities with high interpersonal communication.

**Conclusion:**

Two intervention implications include the need to (a) consider creative ways of incorporating interpersonal communication as a means of amplifying public health intervention effects (also known as “buzz marketing”), and (b) incorporate gender norms into intervention strategies by, for example, tailoring messages differently for those holding equitable (vs inequitable) gender norms.

## Introduction

One in three women have been subjected to physical and/or sexual violence by an intimate partner, a non-partner, or both at least once in their life.^1^ The United Nations (UN) characterizes gender-based violence as an act leading or likely to cause physical, sexual, or psychological harm or suffering to women. This includes threats of such acts, coercion, or unjust deprivation of freedom, whether experienced in private or public life.^2^ Women and girls from low- and middle-income countries are disproportionately affected by gender-based violence.^3^

Gender-based violence has been increasingly recognized as a critical public health issue by the World Health Organization.^4^ It hinders the attainment of equality, progress, and overall well-being. In addition, rates of depression, anxiety, sexually transmitted infections, and unplanned pregnancies are higher in women who have experienced such violence, with lasting effects even after the violence has ended.^1^

In India, as around the world, gender-based violence continues to challenge the livelihood and well-being of women, with the most recent National Family Health Survey (NFHS-5) reporting that 29.3% of ever-married women aged 18–49 years have experienced spousal violence (which is but one subset of gender-based violence) at some point in their lifetime.^5^ Women living in rural areas bear the highest brunt of this violence, with 31.6% of them reporting spousal violence, compared to 24.2% of women in urban areas.^5^ Indeed, even with the presence of laws, programs, policies, and interventions aiming to prevent gender-based violence, its prevalence in India remains unacceptably high.^6^

Unequal power dynamics between men and women due to a patriarchal societal structure is seen as a major driving force behind the high rates of gender-based violence in India.^7^ Endemic violence poses a risk to all women, but the burden falls most squarely on those already experiencing other social, economic, and health disadvantages. Risk factors for gender-based violence include low literacy levels, poverty, unemployment, mental disorder, exposure to previous parental violence, age, lack of community and legal sanctions against violence, and gender norms promoting such violence.^8,9^ In general, long-established unequal gender standards fuel the widespread occurrence of gender-based violence. It is, therefore, incumbent upon those seeking to reduce gender-based violence to consider both risk factors and gender norms in designing interventions. In this paper, based on a secondary data analysis of a project that sought to reduce anemia in India (described subsequently), we ask whether an unintended byproduct of the gender-sensitive approach adopted by the intervention was the reduction of gender-based violence, as assessed by people’s intention to intervene.

Based on our formative assessment, where the issue of gender-based violence came up spontaneously, without our prompting, we came to appreciate that the relationship between gender and gender-based violence is especially critical in India. Indeed, the Global women’s Health Index, based on a recent longitudinal global Gallop survey, deemed India “a country in crisis” and found that between 2020 and 2021, Indian women’s score on the overall Index declined—the biggest drop for any country in the world.^10^ It is perhaps time to approach this issue with a somewhat different lens.

One such lens might be to focus on understanding how prevailing cultural norms overlap with gender-specific norms to perpetuate violence. For example, when professional and social roles are gendered and immutable—when men’s and women’s roles in society are fixed, without interchangeability—norms that empower one gender over another may be related to acts of violence. In this paper, we ask how an understanding of gender norms may play a part in addressing this recalcitrant issue.

### Role of gender norms and social norms

Both the continued perpetration of gender-based violence and the low reporting rate are in no small part due to deeply ingrained detrimental social and gender norms, entrenched stigma, and the fear of reprisals.^11^ Gender norms allow for the physical, sexual, and emotional subjugation of women and are defined as “a subset of social norms that describe how people of a particular gender are expected to behave, in a social context.”^12^ In addition, gender norms are integrated within institutions, strengthened through social interactions, and can result in significant negative health outcomes.^13^

Inequitable gender norms, operating both officially and unofficially, limit women’s independence and access to resources and perpetuate human rights violations, such as gender-based violence.^14^ More specifically, patriarchal and “hyper-masculine” cultural beliefs are associated with gender-based violence.^15^ Stereotypical expectations regarding gender roles, where men predominantly serve as primary providers and women manage household responsibilities, place women in a position of reliance, complicating their ability to leave an abusive relationship.^16,17^

We should note that the use of “gender norms” here and in the literature appears somewhat at odds with the specific types of social norms articulated in the focus theory of normative conduct^18^ and the theory of normative social behavior (TNSB).^19^ In these theories norms are described as descriptive and injunctive.^20,21^ The underling idea is that, to the extent that human behavior is guided by social norms, such influences can be partitioned into those emanating from beliefs that many others engage in the behavior (descriptive norms) or that others expect them to engage in the behavior themselves (injunctive norms). These ideas are also applicable to understanding normative influences within the context of gender-based violence.

In societies where gender-based violence is perceived to be more common (descriptive norms) or where a substantial proportion of the population condones abuse (injunctive norms), women’s risk of experiencing, tolerating, or rationalizing gender-based violence tends to be higher.^22,23^ Despite the role these two dimensions of social norms play in gender-based violence, measures of gender norms have not traditionally incorporated them. The exception is the G-NORM scale, one used in this paper and subsequently described in the Method section.

### Norms and communication in the RANI project

Communication plays a critical role in normative influences; it is through communication that people come to understand acceptable behavioral norms in a given cultural environment.^24^ This has been demonstrated in understanding how alcohol norms are spread among undergraduate students in college^25^ or how norms around modern contraception use are shared among the urban poor in India.^26^

Public health interventions have often relied on communication strategies to promote dialog around an array of health-related topics.^27–30^ One such intervention, the Reduction of Anemia through Normative Innovations (RANI) Project, actively sought to understand the role interpersonal communication might play in perpetuating gender-based violence.^31^ By gathering individuals in the community on a regular basis, the intervention propagated social connections, resulting in greater interpersonal communication. The intervention also aimed to foster an environment where discussions on gender-based violence could evolve naturally within the community. What is unknown, however, is the extent to which interpersonal communication, gender norms, and the intervention independently or jointly affect gender-based violence

The relationship between interpersonal communication and gender-based violence attitudes and behaviors is likely complex. On the one hand, with increasing levels of interpersonal communication, people may acquire greater affinity for each other and thus be more open or willing to shelter each other from harm, including from gender-based violence. Interventions that promote such discussion in the community could result in reducing support for gender-based violence and promoting people’s intentions to intervene when they witness a violent act. On the other hand, if prevailing gender norms are strong and supportive of maltreatment of women, higher levels of interpersonal communication may be counterproductive; they could further perpetuate negative beliefs and behaviors because interpersonal communication would then serve to amplify the existing negativity. We have seen this effect, often unintended,^32^ of interpersonal communication in other health domains, including in perpetuating stigma^33^ and in promoting pro-drug use attitudes and intentions,^34^ leading Southwell and Yzer to note that “discussion can, at times, be an uncooperative partner for campaign planners.”^35^

Also of interest in this paper is how an intervention designed to reduce anemia may be linked with gender-based violence. Our underlying theory of change posits that the key component of the intervention, which brought people together in the village weekly to discuss a host of issues (including, diet, anemia, iron folic acid, gender dynamics, and relationships between men and women in the community), may have enhanced trust among participants, facilitated the discussion of violence in the community, and thus may have improved women’s intention to act when they saw someone in their community being subjected to violence.

Our research questions are:

**RQ1**. How do descriptive and injunctive gender norms, as assessed in the G-NORM scale, affect attitudes toward violence against women and intentions to intervene?
**RQ2**. What is the relationship between interpersonal communication on the one hand, and attitudes toward violence against women and intentions to intervene, on the other?
**RQ3**. Did the RANI Project reduce support for gender-based violence and intentions to intervene, despite the fact that the project itself focused more on anemia and women’s health?

## Methods

The reporting of this study conforms to the CONSORT 2022 Extension for reporting of randomized trials with post-hoc outcomes,^36^ and the checklist is attached to this article. Data for this study come from the Reduction in Anemia through Normative Innovations (RANI) Project, a 3-year randomized controlled trial conducted in Odisha, India to reduce anemia among women of reproductive age. The protocol for the original study^37^ and the primary outcomes in behaviors^38,39^ have been published elsewhere. Ethical approval was obtained in India from Sigma Science and Research (ID 10059/IRB/19-20) and the Indian Council for Medical Research (ID 2018-0921/F1). In addition, IRB approval was obtained from the George Washington University in the United States (IRB #180187).

Our formative assessment had found that harmful norms manifest in four ways: women did not prioritize their own health (over that of other family members), they often lacked autonomy to seek medical treatment on their own, they ate last at mealtimes, and they lacked decision-making authority in the home.^40^ The RANI project thus focused on addressing these broader norms and, more directly, on anemia itself, through the following means:

- Testing women regularly for their anemia (done through finger-prick blood draws to measure hemoglobin) and providing feedback on what their hemoglobin count revealed and what they could do to prevent and reduce anemia.
- Holding cooking sessions to promote recipes for iron-rich foods.
- Producing and disseminating videos specifically targeting women, as well as their social networks, which included younger children, husbands, mothers-in-law, and others. The videos featured narratives of women who successfully navigated obstacles associated with iron folic acid consumption and social norms.
- Introducing and facilitating the playing of games that involved both men and women from the community to illustrate how gender roles and gender norms helped perpetuate harmful practices in the community.

The study randomized village clusters into treatment and control arms, keeping a two- to three-village cluster as a buffer to minimize contamination. Activities noted above were run in the treatment arm, whereas no activity was conducted in the usual-care control arm. The data collection process and procedures were identical in both arms.

Research assistants from the same state (Odisha). who spoke the local language, received training in human subjects research, and they worked in four-person teams to collect data through one-on-one interviews. Although the RANI Project collected longitudinal data from control and treatment communities, this paper uses only the cross-sectional data collected at the end of the project because the module on gender-based violence was only included then.

Questions were formulated in English, translated into Odia by local translators, and field tested in the community. The translated versions underwent further scrutiny in our research training workshop, where interviewers and their supervisors (who speak English) provided input on the cultural equivalence of the various items and their suitability for the local context.

### Inclusion and exclusion criteria

Inclusion criteria included that women had to be between 15 and 49 years old, must have been a resident of the village, able to speak Odiya, and (at baseline) must have had no intention of leaving the village in the subsequent 2 years. Women who did not meet these criteria were excluded.

### Measures

#### Demographic variables

For use as control variables, we assessed age, marital status, education (as number of years of schooling), whether the participant was a member of a tribe (which is a protected class in India), and parity (number of children).

#### The G-NORM scale

The G-NORM scale^41^ is a set of theory-based measures that assess both descriptive norms and injunctive norms.^18^ Informed by the focus theory of normative conduct^18^ and the theory of normative social behavior,^19^ it also incorporates Connell’s theory of gender and power.^42^ This last theory considers gender-based division of labor; division of power, control and authority; and the structure of cathexis, which highlights relationships between men and women precipitated through their gender roles. The G-NORM scale was first formulated with data from India^41^ and since has been validated from studies in Nepal^12^ and Uganda.^43^ Following the G-NORM scale’s distinction between descriptive and injunctive norms, we assessed these two dimensions separately, as described below.

#### Descriptive gender norms

Descriptive gender norms were assessed through nine questions that used the same stem “In most families you know,” followed by specific gender roles, decision-making power, and behaviors. For example, an item on gender roles read “In most families you know, taking care of children is only the woman’s job.” One example focusing on decision-making read, “In most families you know, only men make decisions about household income and expenses.” Finally, a question focusing on specific behaviors read “In most families you know, women eat last, after all the family members have eaten.” Responses were recorded on five-point scales ranging from *strongly disagree* (coded as 1) to *strongly agree* (coded as 5). Descriptive gender norms were calculated as the average across the nine items (α = 0.82, *M* = 4.17, SD = 0.60). Thus, higher scores signify stronger beliefs that others engage in gender-stereotypical beliefs; put another way, higher scores represent inequitable gender norms.

#### Injunctive gender norms

Injunctive gender norms used the same format and structure as the descriptive gender norms, using the same nine items, with the difference being that the stem included the word *should*. This signified the injunction, as opposed to just the prevalence of the behavior, as was the case with descriptive norms. So, for example, one of the questions read “Most families you know believe that it *should* be a woman’s job to take care of the children.” Responses across the nine items were averaged into an index (α = 0.81, *M* = 3.71, SD = 0.78). Higher scores on this scale signify greater level of agreement that others think the gender-stereotypical roles should apply; put another way, higher scores imply greater inequitable norms.

#### Interpersonal communication

Following Ganjoo et al.,^31^ interpersonal communication was assessed through three questions that measured the frequency with which women talked with their family, people in their community, and members of women’s groups about the lives of other women in the community. An example item read “You often talk to your family about the health of the women in your family.” Responses, recorded on five-point scales, were averaged into an index (α = 0.65, *M* = 4.16, SD = 0.66). Higher scores signify higher levels of interpersonal communication

#### Intervention effect

The intervention effect was assessed simply as whether the respondent resided in the control arm or the treatment arm. The treatment arm received the RANI intervention, whereas the control arm was held as “usual care,” where no intervention activities were undertaken.

#### Support for gender-based violence

Support for gender-based violence was assessed through five questions that asked if it was OK for a husband to beat his wife under various circumstances, including if she burns the food, is disrespectful to her mother-in-law, and does not keep the house neat and clean. Two other items included statements that women sometimes deserve to be beaten and men have a right to discipline their wives by beating them. We created an index by averaging across the five items (α = 0.70, *M* = 1.28, SD = 0.59), such that higher scores signified greater support for gender-based violence.

#### Intention to intervene

Intention to intervene refers to people’s willingness to stop or interrupt an incident of a gender-based violence that they witness. It was assessed through two questions. The first question asked if the participant saw a man beating a woman, whether she would ask him to stop. The second question asked if the participant saw a man beating a woman, whether she would distract the man so that he stops beating her. Both responses were coded on two-point scales (0 = *no* and 1 = *yes*) and added together into an index (*M* = 0.95, SD = 0.19). We are not reporting the reliability statistic of this measure because we see the two behaviors as being graduated in difficulty in the sense that women may feel comfortable distracting the man, whereas they may not in asking the man to stop. Thus, higher scores mean that women are willing to do more of the underlying behaviors.

#### Sample size considerations

The sample included in this study was based on the parent trial’s power calculations. Given the secondary analysis done in this study, using the same dataset, a separate power calculation was not performed.

#### Statistical analyses

Means and Chi-squares were used to note differences at baseline on key indicators. Pearson correlations described the bivariate relationships among variables, and linear multiple regressions were used to assess study outcomes.

## Results

### Description of the sample

Table 1 shows the distribution of women in both control (*n* = 1894) and treatment (*n* = 1903) arms. Because women were randomized to control and treatment arms, the two groups had similar characteristics. The only notable difference between the control and treatment arms was the tribal status, with the treatment arm containing a smaller proportion of women (24.7%) who belonged to a tribal group, as compared to those in the control arm (31.4%), χ^2^ (1, *N* = 3797) = 22.4, *p* < 0.001. About a quarter of the sample was between 19 and 25 years old, and another third of the women were between 30 and 40 years old.

**Table 1.** Control (*n* = 1894) and treatment (*n* = 1903) arms from the RANI Project.

	Control	Treatment	*χ* ^2^	*p-*Value
Age			4.80	0.31
<19 years	9.2%	7.7%		
19–25 years	27.0	26.0		
25.1–30 years	19.2	19.3		
30.1–40 years	29.7	31.2		
40 years	14.8	15.8		
Married	79.4	80.9	1.51	0.22
Education			3.25	0.35
None	18.3	19.4		
Up to 6 years only	29.3	27.1		
Up to 10 years only	40.1	41.8		
10 years	12.4	11.7		
Member of a tribe	31.4	24.7	22.4	<0.001
Number of children			3.23	0.36
0	24.9	22.6		
1	20.4	20.9		
2	33.2	34.8		
≥3	21.4	21.7		

*Note.* Chi-square compares control-treatment arm differences.

Most women in our sample (approximately 80%) were married, and close to 20% of the sample had had no formal education. A third of the women had two children and another 22% had three or more children.

### Preliminary analyses

Table 2 shows the correlations among variables used in this paper; those above the diagonal pertain to control and those below the diagonal to treatment arms. Though neither anticipated nor hypothesized, we observe more significant associations in the control arm (35 statistically significant associations) than in the treatment arm (27 associations). In the Discussion section, we offer some possible explanations for and implications of this finding for future inquiry.

**Table 2.** Correlations among key variables in control (above the diagonal) and treatment (below the diagonal) arms from the RANI project.

	(1)	(2)	(3)	(4)	(5)	(6)	(7)	(8)	(9)	(10)
(1) Age	1.00	0.34***	−0.54***	−0.05*	0.60***	0.06**	0.11***	0.21***	0.08***	0.05*
(2) Married	0.34***	1.00	−0.21***	−0.02	0.46***	0.03	0.05*	0.22***	0.02	0.06**
(3) Education	−0.55***	−0.18***	1.00	−0.18***	−0.45***	−0.19***	−0.24***	−0.09***	−0.19***	−0.01
(4) Tribe member	−0.04	0.00	−0.23***	1.00	0.07**	−0.01	0.04	−0.09***	0.11***	−0.05*
(5) No. of children	0.60***	0.50***	−0.44***	0.07**	1.00	0.11***	0.11***	0.20***	0.12***	0.06**
(6) G-NORM, descriptive	0.07**	0.04	−0.22***	0.05*	0.09***	1.00	0.46***	0.21***	0.06**	−0.03
(7) G-NORM, injunctive	0.11***	0.06**	−0.23***	−0.02	0.10***	0.61***	1.00	0.01	0.23***	−0.14***
(8) IPC	0.13***	0.18***	−0.02	0.01	0.14***	0.21***	0.08**	1.00	0.01	0.11***
(9) Support for GBV	−0.04	−0.01	−0.17***	0.12***	0.04	0.13***	0.21***	−0.12***	1.00	−0.03
(10) Intention to intervene	−0.03	0.04	0.08***	−0.04	−0.02	0.01	−0.03	0.02	−0.01	1.00

*Note.* G-NORM: gender norm scale; IPC: interpersonal communication; GBV: gender-based violence.

Correlations in the control (treatment) arm are shown above (below) the diagonal.

* *p* < 0.05, **p < 0.01, ***p < 0.001.

Of particular interest in this paper are the two G-NORM variables and interpersonal communication as predictors, on the one hand, and support for gender-based violence and intention to intervene as the dependent variables, on the other hand. Injunctive G-NORM was negatively associated with intention to intervene (*r* = −0.14, *p* < 0.001) in the control arm, but not in the treatment arm (*r* = −0.03, *p* > 0.05). This difference in these two correlations was significant, *z* = 4.32, *p* < 0.001 (see Appendix 1 for the formula used to calculate the *z*-score). The correlation between injunctive G-NORM and support for gender-based violence in the control arm (*r* = 0.23, *p* < 0.001), but this was not different from the corresponding correlation (*r* = 0.21, *p* < 0.001) in the treatment arm (*z* = 0.64, *p* > 0.05), even though both correlations were themselves significant.

Descriptive G-NORM was associated with support for gender-based violence in the control arm (*r* = 0.06, *p* < 0.05) and in the treatment arm (*r* = 0.13, *p* < 0.001), and these correlations were significantly different from each other, *z* = 2.18, *p* < 0.01. Descriptive G-NORM was not associated with intentions to intervene in either the control or treatment arm.

The association between interpersonal communication and intention to intervene in the control arm (*r* = 0.11, *p* < 0.001) was significantly greater (*z* = 2.78, *p* < 0.01) than in the treatment arm (*r* = 0.02, *p* > 0.05). The association between interpersonal communication and support for gender-based violence was significantly greater (*z* = 4.02, *p* < 0.001) in the control arm (*r* = 0.01, *p* > 0.05) than in the treatment arm (*r* = −0.12, *p* < 0.001). Interpersonal communication was associated with injunctive G-NORM in the treatment (*r* = 0.08, *p* < 0.01) but not in the control (*r* = 0.01, *p* > 0.05) arm, and this difference was statistically significant (*z* = 2.16, *p* < 0.05).

In both control and treatment arms, support for gender-based violence was not associated with intention to intervene. The association between demographic variables and intention to intervene were generally small. Education in the treatment arm was associated with intention to intervene (*r* = 0.08, *p* < 0.001), whereas this association was not observed in the control arm (*r* = −0.01, *p* > 0.05), and the two correlations were significantly different from each other (*z* = 2.78, *p* < 0.01). Education was negatively associated with support for gender-based violence in both the control (*r* = −0.19, *p* < 0.001) and treatment (*r* = 0.17, *p* < 0.001) arms, and these two correlations were not different from each other (*z* = 0.64, *p* > 0.05).

### Research question 1

Our first research question focused on the relationship between descriptive and injunctive gender norm, on the one hand, and attitudes toward gender-based violence and intention to intervene, on the other. Results from multivariate regression equations, controlling for demographic variables, are shown in Table 3.

**Table 3.** Multivariate predictors of attitudes supporting violence against women and intentions to act.

	Support for GBV			Intention to intervene		
	β_a_	CI	Δ*R*²	β_a_	CI	Δ*R*²
Age	−0.04	(−0.08, 0.01)		0.01	(−0.04, 0.05)	
Married	−0.03	(−0.07, 0.00)		0.04*	(0.00, 0.08)	
Education	−0.13***	(−0.17, −0.09)		0.02	(−0.02, 0.06)	
Tribal member	0.08***	(0.04, 0.11)		−0.04*	(−0.07, −0.01)	
No. of children	0.04*	(0.00, 0.09)		0.02	(−0.03, 0.06)	
DGN	−0.04*	(−0.08, 0.00)		0.04*	(0.00, 0.08)	
IGN	0.21***	(0.18, 0.25)		−0.12***	(−0.15, −0.08)	
IPC	−0.04*	(−0.08, −0.01)		0.06***	(0.03, 0.10)	
Intervention (IT)	−0.04**	(−0.07, −0.01)		0.05**	(0.01, 0.08)	
*R*-squared			0.079***			0.022***
IT × IPC	−0.14***	(−0.20, −0.08)	0.006*	−0.11*	(−0.21, −0.01)	0.001*
DG*n* × IGN_b_	0.05**	(0.01, 0.08)	0.002**	N/A	N/A	
IT × IGN_b_	N/A	N/A		0.14**	(0.06, 0.22)	0.003**

*Note.* GBV: gender-based violence; DGN: descriptive gender norms; IGN: injunctive gender norms; IPC: interpersonal communication; N/A: not applicable as the variable was not entered into the equation.

a Standardized beta coefficient from regression equations.

b Interaction terms entered one at a time to avoid multicollinearity. Only significant interactions are shown.

* *p* < 0.05, **p < 0.01, ***p < 0.001.

Findings revealed that inequitable descriptive gender norm was negatively associated with support for gender-based violence (β = −0.04, *p* < 0.05). Descriptive gender norm was also associated with intention to intervene (β = 0.04, *p* < 0.05). Thus, those who believed that others engaged in gender-stereotypical behaviors were less likely to support gender-based violence and more likely to intervene (though the effects were small).

The relationship between injunctive gender norm and attitude toward gender-based violence was significant (β = 0.21, *p* < 0.0001), as was its relationship with intention to intervene (β = −0.12, *p* < 0.001). Thus, those who believed others supported gender-stereotypical beliefs were themselves more likely to support gender-based violence and less likely to intervene.

### Research question 2

Our second research question explored the role of interpersonal communication in people’s support for gender-based violence and their intention to intervene. We found a small but significant relationship between interpersonal communication and support for gender-based violence (β = −0.04, *p* < 0.05) and significant relationship between interpersonal communication and intention to intervene (β = 0.05, *p* < 0.01). Thus, those who engaged in discussion in their communities were less likely to support gender-based violence and more likely to intervene.

### Research question 3

Our third research question asked whether the intervention resulted in lower levels of support for gender-based violence. As shown in the table, we observed a weak, though significant, relationship (β = −0.04, *p* < 0.05), such that those in treatment communities were less likely to harbor supportive beliefs about gender-based violence, as compared to those in control communities. We also observed a similar outcome with regard to intention to intervene. Those in treatment communities were significantly more likely to express intentions to intervene, as compared to those in control communities (β = 0.05, *p* < 0.01). Overall, our model explained 8% of the variance in attitudes toward gender-based violence and 2% of the variance intention to intervene.

### Interaction effects

In the absence of a priori hypotheses about interaction effects, we tried each two-way interaction pair (formed among the two gender norm components, the intervention effect, and interpersonal communication) as a potential interacting variable for each of the two dependent variables. As shown in Table 3, two of the interactions were significant for the first dependent variable (support for gender-based violence), and two were significant for the second dependent variable (intention to intervene). To avoid multicollinearity, we entered only one interaction term at a time, removing it before testing the subsequent interaction.

**Figure 1.**
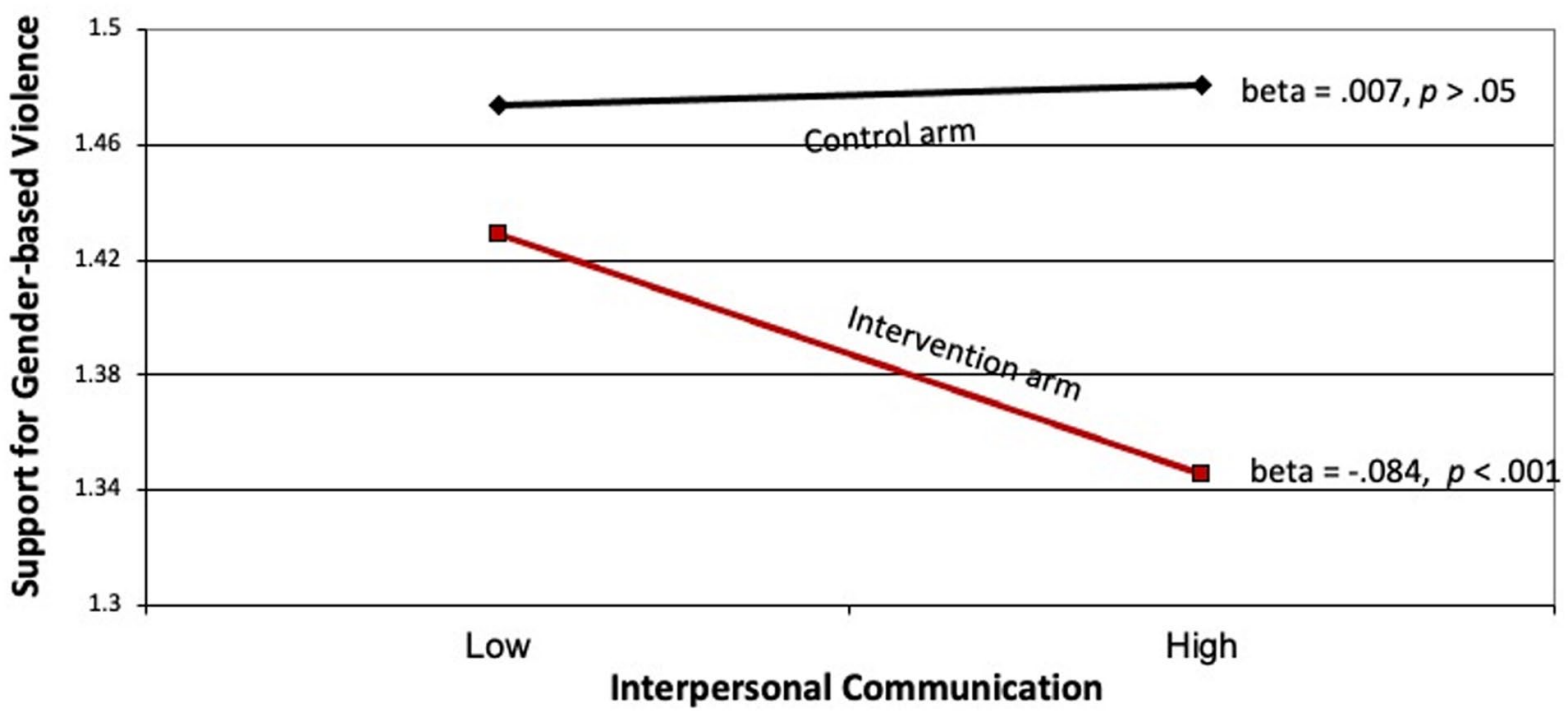
Relationship between interpersonal communication and support for violence in control and treatment arms.

We found a significant interaction effect between the intervention and interpersonal communication on the one hand, and support for gender-based violence, on the other (beta = −0.14, *p* < 0.001). This interaction effect, though statistically significant, only explained approximately 0.6% of the variance. Nevertheless, the pattern of the interaction is shown in Figure 1. The first thing to note in this figure is that, overall, support for gender-based violence was significantly higher in the control arm as compared to the intervention arm. This main-effect, however, has to be interpreted in the context of the interaction effect, which shows that interpersonal communication in the control arm did not differentially impact support for gender-based violence. In the intervention arm, however, greater interpersonal communication resulted in significantly lower levels of support for gender-based violence. The pattern of the interaction also showed that when interpersonal communication was low, the difference in support for gender-based violence between the control and intervention arm was small. This difference was significantly enhanced when interpersonal communication was high.

**Figure 2.**
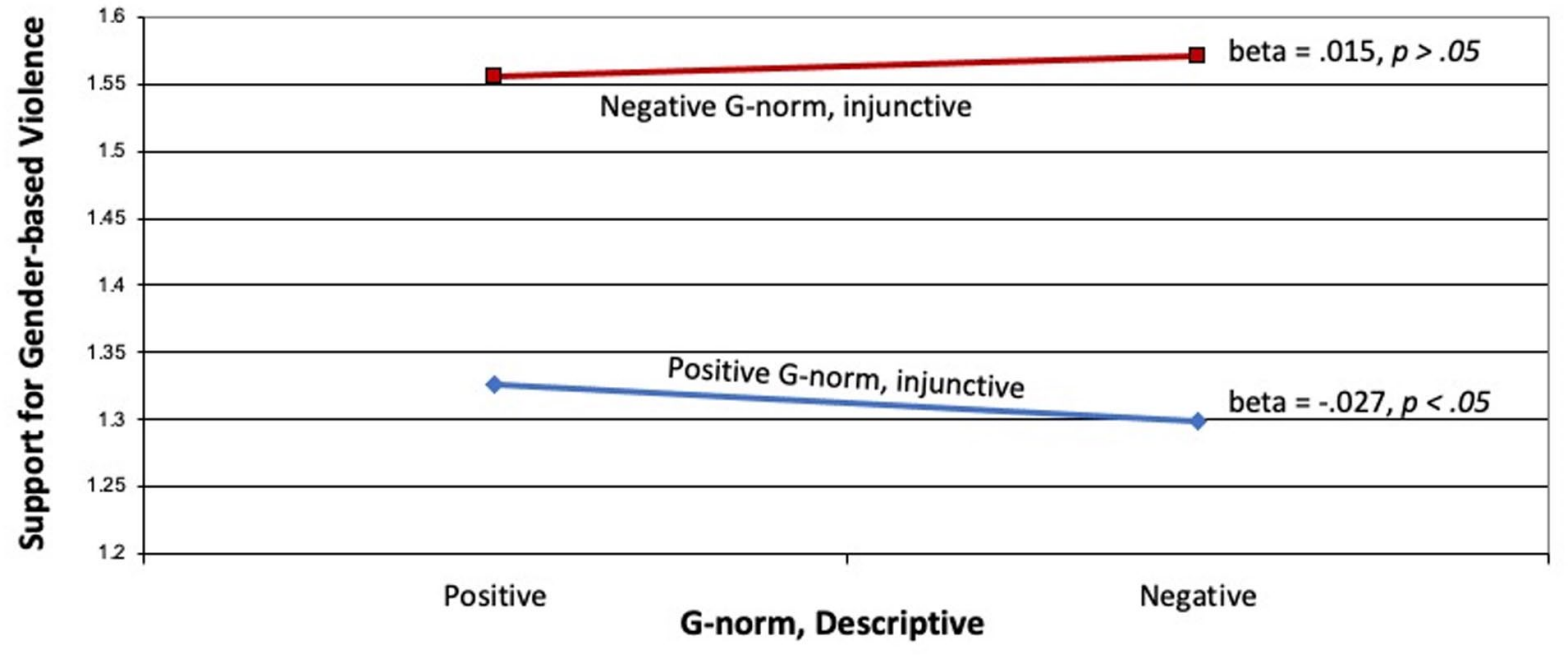
Effects of negative (i.e. more inequitable) descriptive and injunctive norms on support for violence.

As shown in Figure 2, we observed a significant interaction effect between the two gender norm components and their effect on support for gender-based violence (beta = 0.05, *p* < 0.01). As shown in the figure, the combination of negative injunctive and descriptive norms resulted in the highest level of support for gender-based violence. Least support for gender-based violence was observed not when both normative components were positive, but when a negative descriptive norm was paired with a positive injunctive norm.

**Figure 3.**
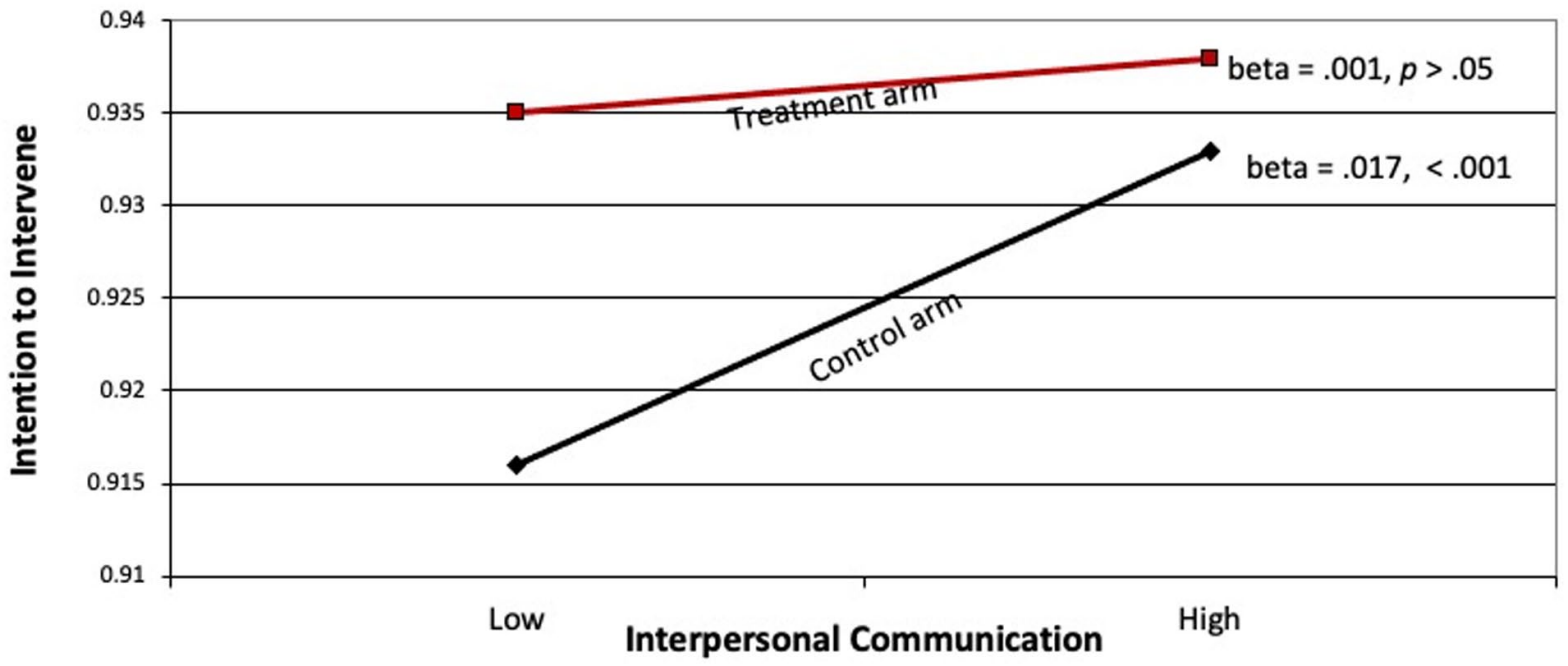
Relationship between interpersonal communication and intention to intervene in control and treatment arms.

We observed a significant interaction effect between the intervention and interpersonal communication, on the one hand, and support for gender-based violence, on the other hand (beta = −0.02, *p* < 0.05). The nature of the interaction is shown in Figure 3. As shown in the figure the treatment arm had significantly higher levels of intentions, as compared to the control arm. Furthermore, in the treatment arm discussion did not result in further enhancing intentions to act; In the control arm, however, greater discussion resulted in greater intentions.

**Figure 4.**
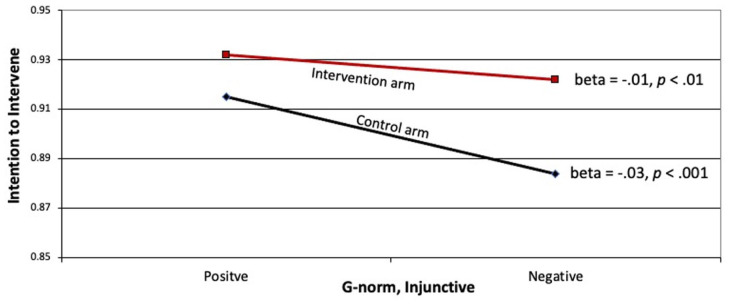
Relationship between negative (inequitable) injunctive norms and intentions to intervene in treatment and control arms.

The intervention and injunctive norm interacted to affect intention to intervene (beta = 0.14, *p* < 0.01). The nature of the interaction, shown in Figure 4, reveals that intentions were higher in the intervention arm, as compared to the control arm. Furthermore, negative injunctive norms resulted in reductions in intentions to intervene in both arms, but the reduction was significantly greater in the control arm as compared to the treatment arm.

## Discussion

In this paper, we sought to lay out the initial knowledge base to understand how prevailing norms in society, interpersonal communication in the community, and an external intervention jointly affect attitudes and behaviors toward gender-based violence. Instead of focusing on a behavior-specific social norm, the G-NORM scale adopts a more expansive view, which includes separate dimensions of descriptive and injunctive norms but also assesses existing power dynamics and gender roles. It is an indication of people’s beliefs about gender roles subscribed to (injunctive) and practiced by (descriptive) others in their community.

A number of interesting findings emerged from our study. Before discussing the gender roles aspect of our findings, we first focus on the nature of correlations we observed in control versus treatment arms. Overall, we saw many significant zero-order correlations in the control arm, as compared to the treatment arm. It is instructive to recall that data for this study were collected at the end of a 3-year project in which people in the treatment arm had had extensive interactions with community members, with the introduction of anemia as a health topic. One of the topics addressed in the intervention was highlighting the differential roles that men and women play in the community and how those roles may impact women’s health. There were also weekly hemoglobin testing sessions, after which women were advised about their anemia readings, methods of prevention, and the necessity of taking and folic acid.

In the larger sense, there was a notable level of disruption in the treatment, but not in the control, arm of the study. We suspect that it is through these disruptions that existing correlations among variables were attenuated: The external intervention caused a disruption in the existing homeostatic balance in the community. While this finding seems a bit esoteric, it provides initial insights about the process of change: that disturbance in the status quo is a first step in improving community-level behaviors. This finding needs to be corroborated through other future studies, but if it holds, it means that interventions need to pay attention to addressing disturbances that are likely to occur at the initial stages of community change. Anticipating those disturbances, discussing with the community about means of addressing them, and proactively taking steps to reduce possible damages seem like prudent steps for community-based interventions to adopt.

We found that injunctive gender norm was negatively associated with intention to intervene in the control arm (meaning that those who believed most others supported gender-stereotypical beliefs were themselves less likely to intervene), and this relationship was weaker in the treatment arm. One explanation for this finding is that, in the absence of an intervention, people are likely to resort to their existing (in this case, stereotypical) beliefs. Hence those who harbored negative beliefs about gender roles would be less likely to interrupt or get in the way of the gender-based violent action they witness. It could be that the intervention gave people reason to question the status quo, an example of the disturbances in the homeostatic balance noted above, and thus their existing attitudes and beliefs about gender roles were less predictive of their intention to act, something we observed in the intervention arm.

We found higher levels of support for gender-based violence in the control arm, relative to the intervention arm. This main effect, however, has to be interpreted in the context of the interaction, shown in Figure 1. In the control arm, the higher level of support for gender-based violence was not affected by interpersonal communication. In the intervention arm, however, higher levels of interpersonal communication resulted in lower levels of support for gender-based violence. One interpretation of this finding is that, in control communities, interpersonal communication was simply promoting the status quo, and hence attitudes did not differ by level of interpersonal communication. In treatment communities, people had had exposure to gender-related issues introduced by the campaign, and hence greater interpersonal communication served to change existing attitudes. This is in line with Jeong and Bae’s meta-analysis^44^ showing that intervention-generated interpersonal communication can promote intervention outcomes.

Between the two dimensions of gender norms articulated in the G-NORM scale, it appears that injunctive norms are more instrumental in affecting both support for gender-based violence and intentions to intervene. As shown in Figure 2, those with stronger (and thus more negative) injunctive beliefs were significantly more likely to express support for gender-based violence, as compared to those with weaker (or more positive) injunctive beliefs. We also saw a mild interaction between the two types of norms such that the most support for gender-based violence was observed when both norms were strong. When people held weaker injunctive norm beliefs (i.e. their beliefs were more positive), greater descriptive norms resulted in lower levels of support for gender-based violence. The overall effect size is rather small, so this finding has to be taken with much skepticism; nevertheless, it appears that having positive injunctive norms can overcome some of the negativity brought about through stronger descriptive norms.

We observed a significant interaction effect between interpersonal communication and the intervention on intention to intervene, as shown in Figure 3. This is in line with Southwell and Yzer’s identification of interpersonal communication as a moderator of intervention effects.^35,45^ Apart from the main-effect of the intervention (intention to intervene was greater in the treatment than in the control arm), the pattern of the interaction showed that personal communication boosted intentions to act in both communities, though it was stronger in the control arm of the study. Put another way, when interpersonal communication was low, the intervention had an outsized effect; when interpersonal communication was high, the effect of the intervention was less pronounced, likely because discussions served as another source of information and influence. This mirrors findings reported by Rimal et al. in their intervention in Malawi: when people did not engage in interpersonal communication, the gap in knowledge and HIV testing behavior between those exposed and not exposed to the campaign, which was considerable, was significantly reduced when interpersonal communication was high.^30^

This finding likely illustrates the power of interpersonal communication to serve as an important channel of influence, particularly when the goal is to reduce disparities in knowledge and information. The knowledge gap hypothesis^46^ posits that, when interventions appear in a community, they can exacerbate disparities in the community. Our findings indicate that one way to attenuate those disparities would be to promote interpersonal communication, as has also been reported elsewhere.^47^

Interpersonal communication as a distinct intervention strategy to promote behavior change may not be used often in public health, but it is an integral part of commercial marketing. Indeed, *buzz marketing* (which refers to “contagious talk about a brand, service, product, or idea,”^48^) is used extensively by commercial producers to sell products by stimulating discussions that are often deliberately seeded.^49^ How such strategies can be ethically used in public health remains a fruitful area for future research.

We found that gender-based violence attitudes and behaviors were not correlated (in both treatment and control arms): those who supported gender-based violence were no more or less likely to harbor intentions to intervene, as compared to those who held negative attitudes toward gender-based violence. This signifies that intentions to intervene are guided by other factors. In our study, variables positively associated with intention to intervene were holding positive injunctive norms, engaging in interpersonal communication, and living in the intervention arm of the study. This raises questions about the efficacy of intervention strategies that seek to change people’s attitudes toward gender-based violence as a means of promoting action. Because attitudes, particularly those entrenched deeply in a community over a long period of time, are often difficult to change, a more effective approach may lie in facilitating changes in behaviors, instead. It could be done by addressing social norms and by modeling appropriate behaviors, which would communicate that others are acting and “you can, too.”

We noted earlier that subscribing to gendered professional roles may be related to gender-based violence. The G-NORM scale assesses people’s beliefs about the prevalence of (descriptive norms) and support for these gendered roles (injunctive norms) in their communities. We did find that inequitable descriptive and injunctive norms were associated with support for gender-based violence, but not with intentions to act. This association was stronger in treatment than in control arms, and it likely means that being exposed to the RANI Project intervention resulted in lower inequitable descriptive norms, which in turn resulted in lower levels of support for gender-stereotypical beliefs.

The relationship between inequitable injunctive norms and support for gender-based violence (*r* = 0.23, *p* < 0.001 in control and *r* = 0.21, *p* < 0.001 in treatment arms) was significantly stronger than that between inequitable descriptive norms and support for gender-based violence (*r* = 0.06, *p* < 0.01 in control and *r* = 0.13, *p* < 0.001 in treatment arms); *z* = 5.36, *p* < 0.001 and *z* = 2.54, *p* < 0.01 for control and treatment communities, respectively. It thus appears that reductions in support for gender-stereotypical beliefs can be achieved through a strategy designed to influence injunctive gender norms than one targeting descriptive gender norms. Whether and to what extent injunctive gender norms are modifiable through an external intervention is a question we cannot address in this paper; we hope future research will take this up.

### Limitations

A significant limitation of this study is the small effect sizes we are reporting. Because of a large sample size, even small relationships (in the magnitude of *r* = 0.04) appear to be statistically significant. Indeed, our models were able to explain only about 8% of the variance in attitude toward gender-based violence as the outcome and only 2% of the variance in intention to act as the outcome. This likely means the underlying issue is deeply entrenched in the culture and thus less amenable to change in the short-term.

Another limitation is that the RANI Project was designed to promote women’s consumption of iron folic acid; it was not specifically seeking to reduce support for gender-based violence or to promote intentions to intervene. Rather, this domain was a by-product on the intervention path: To change social norms around women’s consumption of iron folic acid, it was also necessary to highlight power imbalances and gender roles. Because reducing gender-based violence was not the central but rather a peripheral campaign goal, it is perhaps not surprising that our effect sizes were rather small. Nevertheless, we uncovered important insights about how gender norms and interpersonal communication can be linked with gender-based violence.

Finally, we developed our own measures of intention to intervene and support for gender-based violence. Though field tested in our study before administering them, that they were not previously validated constitute another limitation.

## Conclusion

The G-NORM scale, which assesses gender norms through both descriptive and injunctive components, appears to be a useful tool with which to study the context in which violence against women is perpetrated and perpetuated. Interventions that promote interpersonal communication are likely to see positive outcomes if gender norms are also targeted for change. We observed distinct effects of interpersonal communication, depending on whether discussion took place among people with weak or strong stereotypical beliefs. In the latter case, when gender-stereotypical beliefs were strong, interpersonal communication could be counterproductive. This is an important learning from this paper.

## Appendix 1

Steps involved in testing the statistical significance of the difference between two correlations

1. Convert the two correlations into *z*-scores:

$$Z_{1}=\frac{1}{2}\;log_{e}\;\frac{1+r_{1}}{1-r_{1}}$$

$$Z_{2}=\frac{1}{2}\;log_{e}\;\frac{1+r_{2}}{1-r_{2}}$$

2. Calculate the standard error (where *N*_1_ and *N*_2_ correspond to the sample size of each group):

$$standard\;error=\;\frac{1}{\sqrt{\frac{1}{N_{1}-3}+\;\frac{1}{N_{2}-3}}}$$

3. Convert to *z*-value:

$$Z-statistic=\frac{Z_{1}-\;Z_{2}}{standard\;error}$$

4. Consult *z* – tables to calculate the significance of the *z*-statistic.
